# Chromosome-level genome assembly of the Australian *Chenopodium trigonon* reveals a new subgenome type

**DOI:** 10.3389/fpls.2026.1841078

**Published:** 2026-06-11

**Authors:** Ashley K. Marcheschi, Karol Krak, Bohumil Mandák, John Sproul, David Jarvis, Eric N. Jellen, Peter J. Maughan

**Affiliations:** 1 Department of Plant and Wildlife Sciences, Brigham Young University, Provo, UT, United States; 2 Department of Ecology, Czech University of Life Sciences, Prague, Czechia; 3 Institute of Botany of the Czech Academy of Sciences, Průhonce, Czechia; 4 Department of Biology, Brigham Young University, Provo, UT, United States

**Keywords:** Australian *Chenopodium*, genome size variation, LTR retrotransposons, polyploid evolution, quinoa relatives, subgenome divergence

## Abstract

The genus *Chenopodium* comprises a diverse assemblage of diploid and polyploid species distributed worldwide, yet Australian taxa remain genomically undercharacterized. Here, we report a chromosome-level genome assembly of *Chenopodium trigonon*, an Australian halophytic tetraploid. Using 23.6 Gb of PacBio HiFi and Hi-C data, we generated an 830.9 Mb assembly with high contiguity (scaffold N50 = 45.9 Mb), 99.2% BUSCO completeness, and near-complete chromosomal representation (n = 18). Comparative synteny, orthology, and repeat landscape analyses were performed to investigate subgenome composition, evolutionary relationships, and genome evolution. Our analyses revealed that *C. trigonon* is an allotetraploid composed of an A subgenome and a subgenome belonging to a deeply divergent G/H-like lineage that we designate as a novel “I” genome, showing pronounced structural divergence relative to the G and H subgenomes. Both subgenomes form sister groups to other members of their respective clades and exhibit extended branch lengths, consistent with an independent polyploidization event relative to *C. sosnowskyi*. Repeat landscape analyses demonstrated that genome size differences between species are largely driven by differential LTR retrotransposon accumulation, with distinct temporal and compositional dynamics across subgenomes. Comparative read-mapping of the Australian octoploid *C. baccatum* further indicated predominant A and I ancestry (putative AAAAIIII), suggesting that an ancestral A–I genomic combination may underlie diversification of *Chenopodium* in Australia.

## 1 Introduction

The continent of Australia contains a unique, highly diverse, and poorly characterized assemblage of diploid, polyploid, woody, and herbaceous plants classified in the circumpolar goosefoot genus *Chenopodium* L. and allied genera of the family Amaranthaceae (Mosyakin and Iamonico, 2017). Jaggi et al. (2025) published whole-genome sequence data for a 12-species pangenome panel designed to capture all the known genomes and subgenomes, A through H (Mandák et al., 2018), of the strict-sense genus *Chenopodium* from the Northern Hemisphere (Fuentes-Bazan et al., 2012, Fuentes-Bazan, Uotila, et al.,2012). Since there are no known, extant diploids composed of genomes C, F, and G, these were characterized within the polyploids *C. strictum* Roth (CCDD), *C. opulifolium* Schrad. Ex Amaranthaceae *senso stricto* ex W.D.J.Koch & Ziz (BBCCFF), and *C. sosnowskyi* Kapeller (AAGG).

Elucidating the evolutionary, systematic, and ecological relationships within *Chenopodium* is of increasing interest due to the growing global popularity of the South American pseudocereal quinoa (*C. quinoa*, 2n = 4x = 36, AABB). In Australia, *Chenopodium* comprises approximately 20–30 taxa (depending on taxonomic treatment) occupying diverse habitats including arid inland regions, saline flats, coastal areas, and disturbed soils. As with quinoa and its Eurasian and Western Hemisphere relatives, this ecologically diverse assemblage may harbor genes valuable for expanding global production of nutrient-dense crops and improving tolerance to aridity, heat, and soil salinity.

*Chenopodium trigonon* Schult., commonly known as fishweed or pigweed, is a prostrate to low-growing halophytic annual of well-drained, disturbed soils native to southeastern Australia and naturalized in the New Zealand archipelago, the Chatham and Kermadec Islands, and South Africa, and it may also occur as an introduced weed elsewhere. The species was previously treated as *Einadia trigonos* (Schult.) Paul G. Wilson. In that treatment, Wilson (1983) recognized three subspecies: *E. trigonos* subsp. *stellulata* (Benth.) Paul G. Wilson, characterized by linear or spathulate tepals and a papillate pericarp; subsp. *trigonos*, with non-linear tepals, a smooth pericarp, and triangular leaves with rounded apices; and subsp. *leiocarpa*, with narrower leaves and acute leaf apices. Although *Einadia* continues to be recognized in some Australian taxonomic treatments (Australian Plant Name Index, APNI), it is not accepted as distinct from *Chenopodium* in New Zealand (New Zealand Flora, NZ Flora), and recent bait-capture data likewise support its placement within *Chenopodium* (Žerdoner Čalasan, pers. comm.). Because *C. trigonon* represents this Australasian lineage and is adapted to saline and disturbed environments characteristic of many Australian chenopods, we selected *C. trigonon* subsp. *stellulata* for genome sequencing. Here we present a reference-quality genome assembly for this taxon as a resource for comparative analyses within a developing *Chenopodium* pangenome framework. Specifically, we (1) generate a high-quality reference assembly using PacBio HiFi and Hi-C data; (2) produce a comprehensive genome annotation supported by transcriptomic evidence; (3) characterize subgenome composition and assign chromosomes using comparative and k-mer based approaches; (4) reconstruct subgenome phylogenetic relationships to infer the evolutionary origins; and (5) examine genome structure, repeat composition, and whole-genome duplication patterns in comparison with related *Chenopodium* species, including the Australian octoploid *C. baccatum.*

## 2 Materials and methods

### 2.1 Plant material and DNA extraction

Seeds were collected and germinated under controlled conditions to obtain fresh, uncontaminated tissue from a single individual for high molecular weight (HMW) DNA extraction. *Chenopodium trigonon* subsp. *stellulata* seeds (BYU 2439; **Supplementary Figure S7A**) were collected in 2024 from Princes Hwy, Swan Reach, Victoria, Australia (-37.823°, 147.855°) (*B. Mandák & K. Krak*, CANB 997549). *C. baccatum* (BYU 2414; **Supplementary Figure S7B**) seeds were collected in 2024 from 0.8 km W from Memorial Rd on Point Peron Rd, Rockingham, Western Australia, Australia (-32.272°, 115.693°) (*K.A. Shepherd & S.R. Willis KS 2008*, PERTH 09668055). Seed was manually scarified and imbibed by shaking for 2 hours at 250 RPM in sterile water, and after sterilization with 20% bleach they were plated on M.S. agar and grown in an incubator (21 °C, 16 hr. daylength; Provo, UT, USA) for two weeks. Seedlings were transplanted into a hydroponics system, with young whole leaf tissue collected for DNA extraction performed as previously described (Jaggi et al., 2025) which uses a Carlson DNA buffer with a QIAGEN® (Hilden, Germany) Genomic-tip 500/G kit.

### 2.2 Genome sequencing and assembly

To obtain PacBio HiFi reads, the HMW DNA was sheared to approximately 17 kb with a Diagenode Megaruptor 3 (Denville, NJ) and converted to SMRTbell libraries with the SMRTbell prep kit 3.0 (Pacific Biosiciences, Menlo Park, CA). The libraries were then size-selected (> 10 kb) on a Sage Science BluePippin (Beverly, MA). Sequencing was performed at the Brigham Young University Genomic and Bioinformatic Center (Provo, UT, USA) on the PacBio Revio platform using the Revio SPQR polymerase kit (PN: 102-182-500). The sample was run for a 30-hour movie time with adaptive loading.

The genome size was estimated by generating a histogram of k-mer counts using Jellyfish v2.2 (Marçais and Kingsford, 2011) using the complete PacBio HiFi data set, which was plotted using Genomescope 2.0 (Ranallo-Benavidez et al., 2020) with k-mer length = 51, ploidy = 4 and default parameters for all other parameters. A primary contig assembly was generated with hifiasm v0.19.9 (Cheng et al., 2021) using the Hi-C data (–h1 and –h2) with the addition of –l 0 and –s 0.4 to preserve subgenome divergence. Assembly errors were identified and corrected using inspector v1.3.1 (Chen et al., 2021). To scaffold contigs, leaf tissue was sent to Phase Genomics (Seattle, WA, USA) for Hi-C library preparation. Libraries were generated using a proximity ligation protocol in which crosslinked chromatin was digested with a four-enzyme cocktail (DpnII, DdeI, HinfI, and MseI), proximity-ligated, and processed into Illumina sequencing libraries (2 × 150 bp). Raw Hi-C reads were mapped to the corrected primary contig assembly using the Arima Mapping Pipeline (Arima Genomics, Document Part Number A160156 v03, Carlsbad, CA, USA) and scaffolded with YaHS v1.2a.1 (Zhou et al., 2023). The scaffolded assembly was then manually curated with Juicebox v2.20 (Durand et al., 2016). Hi-C contact maps generated from the YaHS scaffolding output were visually inspected to identify and correct potential misjoins, orientation errors, and ordering inconsistencies, based on disruptions in expected intrachromosomal interaction patterns. The contact maps exhibited strong chromosome-scale interaction signals with clear diagonal structures and minimal interchromosomal noise, supporting the accuracy of the scaffolding and subsequent manual refinements.

### 2.3 Chromosome and subgenome assignment

Chromosome-length pseudomolecules of *C. trigonon* were mapped to all known *Chenopodium* chromosomes (subgenomes A-H) using minimap2 v2.26 (Li, 2018) with –f 0.02 to exclude the top 2% most frequent minimizers, thereby reducing the spurious alignments from repetitive sequences. The resulting alignments were then visualized using D-Genies (Cabanettes and Klopp, 2018). Chromosome names, orientations, and subgenome designations were assigned based on observed synteny, following the nomenclature established by Jaggi et al. (2025). SubPhaser v1.2 (Jia et al., 2022) was used to verify subgenome and chromosome assignments.

### 2.4 Genome validation

BlobTools v1.1.1 (Laetsch and Blaxter, 2017) was used to screen off-species contaminants among unincorporated contigs using order-level classification with the specification -taxrule bestsumorder. The LTR Assembly Index (LAI) was computed for the *C. trigono*n genome using LTR Retriever v3.0.3 (Ou et al., 2018). Assembly errors were identified using inspector.py from inspector v1.3.1 (Chen et al., 2021) and subsequently corrected with inspector-correct.py. To verify improvement, inspector.py was rerun on the corrected assembly. A k-mer based QV score was computed for the final genome using Merqury v1.3 (Rhie et al., 2020).

### 2.5 Chloroplast assembly and annotation

A *de novo* chloroplast assembly was created using methods previously described by Jarvis et al. (2022) using the *C. quinoa* chloroplast genome (MK159176; Maughan et al., 2019) as the bait sequence. The chloroplast sequence was annotated with GeSeq (Tillich et al., 2017) using the default parameters for a circular plastid. The assembled chloroplast genome was used to identify and remove chloroplast-derived sequences from the nuclear genome assembly and was retained as a separate contig in the final assembly.

### 2.6 Repetitive element composition

Repetitive elements were identified using Earl Grey v6.3.3 (Baril et al., 2024) against the Viridiplantae RepeatMasker database. The TE library was clustered to reduce redundancy, annotations <100 bp were filtered, and Helitrons were detected with HELIANO (Li et al., 2024) with the Dfam partition (Storer et al., 2021) specified for classification. Default settings were used for all other parameters. A soft-masked genome was produced from the final repeat annotations. Filtered repeat annotations were converted to divergence-summed matrices by binning Kimura distances (0–70%) and calculating total base pairs per family per bin. Divergence values were converted to estimated insertion times using the relationship T = K/2r, where r = 1.5 × 10^-8^ substitutions per site per year, a rate derived from LTR retrotransposon evolution in *Arabidopsis thaliana* (Koch et al., 2000) since a lineage-specific substitution rate has not been reported for *Chenopodium*. Repeat landscapes were constructed from isolated Ty1- and Ty3-retrotransposon sequences for the complete assembly as well as the separate subgenomes.

### 2.7 Genome annotation

RNA was isolated from whole leaf, stem, root, and whole seedling tissue using the Direct-zol RNA MiniPrep Plus kit (Zymo Research, R2070). RNA quality was assessed with a Bioanalyzer, and equimolar amounts from each tissue were pooled to synthesize full-length complementary DNA using the NEBNext Single Cell/Low Input cDNA Synthesis and Amplification Kit (E6421L, New England BioLabs). An IsoSeq library was prepared using the SMRTbell v3.0 library preparation kit and sequenced on a single SMRT Cell 8M on a PacBio Sequel II platform at the DNA Sequencing Center at Brigham Young University (Provo, UT, USA).

*Chenopodium trigonon* IsoSeq reads were combined with a genus-level IsoSeq dataset (Jaggi et al., 2025), mapped to the soft-masked genome using minimap2 v2.26 (Li, 2018), and given to BRAKER3 v3.0.7 (Gabriel et al., 2024) as transcript evidence. Gene prediction incorporated protein evidence from Viridiplantae OrthoDB v.11 (Kuznetsov et al., 2023), as well as Caryophyllales sequences from UniProtKB and NCBI RefSeq. BRAKER3 first trained GeneMark-ETP using protein and transcript evidence, then trained AUGUSTUS based on GeneMark-ETP predictions. Gene models predicted by GeneMark-ETP and AUGUSTUS were integrated using TSEBRA (Gabriel et al., 2021).

Functional annotation was assigned by querying predicted protein sequences against the Swiss-Prot database (downloaded July 2023) using BLASTp (e-value < 1.0 × 10^-6^; Ye et al., 2006) and by running InterProScan (Jones et al., 2014) to identify conserved domains and assign Gene Ontology terms (PANTHER v15.0, Pfam v33.1, PIRSF v3.10, PIRSR 2021_02, PRINTS v42.0, and SUPERFAMILY v1.75). Annotation completeness was assessed using BUSCO v5.8.0 (embryophyta_obd10 and eudicots_obd10; Manni et al., 2021).

### 2.8 Genome visualization

The positions of genes in the *C. trigonon* assembly were extracted from the annotation GFF file, and the genomic locations of Ty3-retrotransposons (previously referred to as “Gypsy”) and Ty1-retrotransposons (previously referred to as “Copia”) were obtained from the Earl Grey filtered repeats GFF output (see *Repetitive Element Composition*). Centromeric regions were identified via BLAST (Ye et al., 2006) of the 34 bp (GACTTTCATTTGATTCAATTAGCTTTGTTTGAAT) centromeric satellite subrepeat sequence derived from *C. vulvaria* using TRASH (Wlodzimierz et al., 2023) with an identity ≥ 90% cutoff. TIDK v0.2.63 (Brown et al., 2025) was used to identify telomeric sequences by searching for the canonical Caryophyllales telomeric repeat motif (TTTAGGG). Telomere locations were determined and visualized using the find and plot commands, respectively. The densities of genes, LTR Ty-1 and Ty3-retrotransposons, telomeric repeats, and centromeric repeats were viewed in 500-kb windows for all chromosomes using Circa (http://omgenomics.com/circa).

Synteny between the A and I subgenomes of *C. trigonon* was identified using BLASTp with parameters -max_target_seqs 2 and -evalue 1e-10. These stringent settings were used to retain only the top homologous matches, thereby reducing paralogous and lower-confidence hits and facilitating clearer visualization of syntenic relationships. Synteny between *C. trigonon* and other *Chenopodium* species (*C. sosnowskyi, C. watsonii, C. pallidicaule, C. berlandieri*, and *C. quinoa* for subgenome A; *C. sosnowskyi* and *C. vulvaria* for genomes G, H, and I) was assessed separately for each corresponding subgenome pair using BLASTp with -max_target_seqs 1 and -evalue 1e-10. Syntenic blocks were identified using MCScanX and visualized with SynVisio (Bandi et al., 2022).

### 2.9 Whole genome duplication and phylogenetics

Whole-genome duplication (WGD) events were inferred using WGD v2.0.3 (Chen et al., 2024) for *C. trigonon* and other tetraploid Chenopodium species, including *C. sosnowskyi* (AAGG), *C. strictum* (CCDD), *C. quinoa* (AABB), and *C. berlandieri* (AABB). The paleotetraploid amaranthaceous species *Dysphania ambrosioides* (L.) Mosyakin & Clemants was included for comparison. Paralogous gene pairs were identified via all-versus-all similarity searches, and Ks values were calculated to generate age distributions. Collinearity was used to identify anchor gene pairs, and WGD peaks were estimated from anchor-based Ks distributions using heuristic peak detection with 95% confidence intervals.

A subgenome phylogeny was generated using the multiple sequence alignment workflow implemented in OrthoFinder v2.5.5 (Emms and Kelly, 2015) with the –M msa option enabled. In this approach, orthogroups are inferred and aligned, and gene trees are constructed for each orthogroup using the default OrthoFinder pipeline (FastTree), followed by inference of a species tree using the STAG algorithm and rooted with STRIDE. Bootstrap support values were generated using the internal resampling procedure implemented in OrthoFinder (100 replicates). *Dysphania ambrosiodes* (L.) Mosyakin & Clemants (“epazote”; Frandsen et al., 2025) was included as an outgroup to root the phylogeny. Dysphania R. Br. is a sister genus of *Chenopodium* in *Amaranthaceae*. The tree was visualized using FigTree v1.4.4 (Rambaut, 2018).

## 3 Results and discussion

### 3.1 Genome assembly

DNA sequencing of the *C. trigonon* genome produced 23.640 Gb of PacBio HiFi data with a mean read length of 15.149 Kb or ~ 27.8X coverage. The sequencing data was assembled into a primary draft assembly, consisting of 352 contigs with a total length spanning 839.418 Mb and a contig N50, L50 and GC% of 19.457 Mb, 16, and 35.71%, respectively. Contigs were scaffolded into chromosomes using Hi-C data, generating a chromosome-level assembly consisting of 325 total scaffolds, including 18 large scaffolds (representing 98.3% of the total sequence data) corresponding to the haploid chromosomes (n=18; **Supplementary Figure S1**). Following correction with Inspector (Chen et al., 2021), the genome QV score improved from 53.26 to 56.52. Using the HiFi data we also *de novo* assembled the chloroplast of *C. trigonon*, which spanned a 152,126 bp (**Supplementary Figure S2**) and used that to removed 130 unincorporated contigs that shared ≥ 99% sequence identity with the assembled chloroplast over ≥ 99% of the total contig length. Similarly, we identified and removed three contigs that shared similarity (≥ 99%) to the mitochondria (MK182703) previously reported by Maughan et al. (2019) for *C. quinoa*. 110 unincorporated contigs < 50 Kb were removed. No off-species contaminating contigs were identified with Blobtools (Laetsch and Blaxter, 2017). The final, cleaned genome assembly had 82 scaffolds spanning 830.898 Mb with a N50, L50 and GC% of 45.869 Mb, 9, and 35.66% (**Table 1**). The genome size is consistent with the predicted genome size identified by GenomeScope2 (812 Mb; **Supplementary Figure S3**) and is similar to other allotetraploid *Chenopodium* species, including *C. strictum* (822 Mb) and *C. sosnowskyi* (864 Mb; Jaggi et al., 2025). This estimate is also broadly consistent, although slightly smaller, than an independent flow cytometry measurement (2C = 2.03 pg; P. Trávníček, personal communication, Czech University of Life Sciences), which corresponds to a predicted haploid genome size of approximately ~995 Mb (1 pg = 978 Mb). The discrepancy likely reflects the known tendency of sequence-based assemblies to underestimate genome size due to incomplete representation or collapse of highly repetitive regions, as well as inherent variation in flow cytometry estimates.

**Table 1:** Assembly statistics of the *Chenopodium trigonon* contig, preliminary scaffold, and final scaffold assemblies.

Metric	Contig assembly^a^	Preliminary scaffold assembly^b^	Final scaffold assembly^c^
Total length (bp)	839,418,144	839,951,142	830,898,497
Number of contigs/scaffolds	352	325	82
Largest contig/scaffold (bp)	44,333,959	58,211,597	58,211,597
N50 (bp)	19,457,554	45,869,241	45,869,246
L50	16	9	9
N90 (bp)	8,581,037	40,787,770	40,787,770
L90	39	17	17
Inspector QV Score	–	53.26	56.52
Merqury Completeness (%)	–	–	99.5
Merqury QV Score	–	–	75.69
LTR Assembly Index (LAI)	–	–	19.72
BUSCO Completeness (%)	–	–	99.2

^a^ Primary contig assembly from Hifiasm (Cheng et al., 2021).

^b^ Hi-C scaffolded assembly using YaHS (Zhou et al., 2023).

^c^ Final manually curated genome assembly.

**Figure 1:**
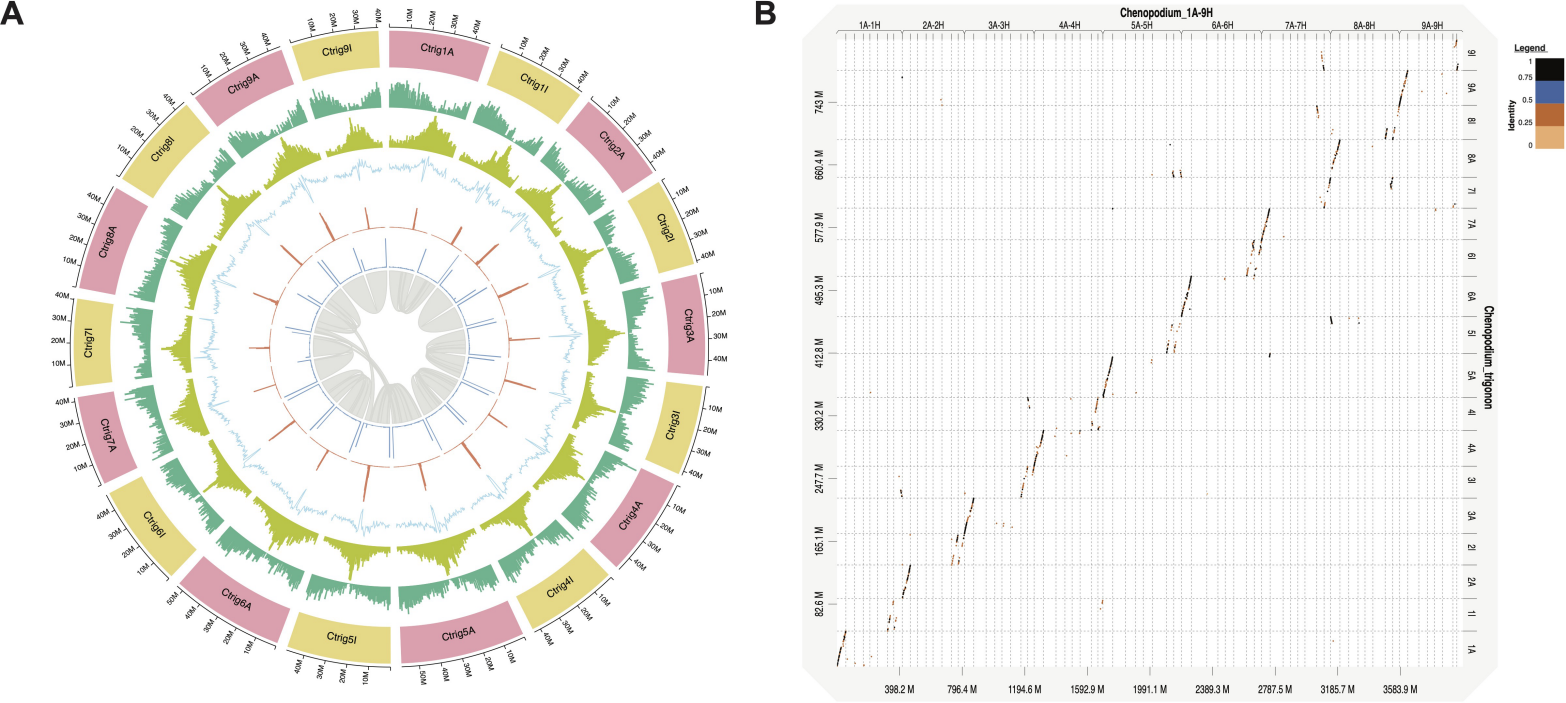
*Chenopodium trigonon* genome features and subgenome relationships. **(A)** Circular representation of the 18 chromosomes in the *C. trigono*n genome assembly, with A subgenome chromosomes shown in pink and I subgenome chromosomes shown in yellow. In order starting from the outside, tracks represent 1) chromosome size (tick marks, 10 Mb), 2) chromosome name, 3) gene density, 4) repeat (LTR Ty1- and Ty3-retrotransposons) density, 5) GC content, 6) centromeric repeat density, 7) telomeric repeat density, and 8) synteny between chromosomes. **(B)** Syntenic dot plot showing homeologous gene relationships between all 18 C*. trigonon* chromosomes (right) and a panel of chromosomes 1–9 from each of the A–H *Chenopodium* subgenomes (top).

The number of contigs per chromosome ranged from one to five, with one chromosome (Ctrig8I) consisting of a single telomere-to-telomere contig, indicating that the chromosome assemblies are largely complete. Indeed, 17 of the 18 chromosomes exhibit a telomeric repeat feature on both chromosomal ends (**Figure 1A**). Similarly, all 18 chromosomes contained a centromeric repeat feature (**Figure 1A**). Benchmarking of universal single-copy orthologs (BUSCO) also indicated that the genome assembly is largely complete with 99.2% and 97.7% of the conserved orthologous genes (COGs) being identified as complete in the embryophyta and eudicot datasets, respectively (**Table 1**, **Supplementary Figure S4**). Not unexpectedly for this polyploid genome, the majority of the BUSCO genes were also duplicated (88.4% and 87.1%, respectively). When BUSCO analyses were performed separately on each of the subgenomes, completeness was substantially reduced relative to the full assembly, as each subgenome contains only a subset of conserved orthologs. This pattern is expected in polyploids as it reflects post-polyploidization processes such as gene loss, fractionation, or neofunctionalization (**Supplementary Figure S4**). We further evaluated genome completeness and base-level accuracy using Merqury (Rhie et al., 2020), a k-mer based approach, which yielded a genome QV of 75.7 and an estimated completeness of 99.5%. Lastly, we assessed assembly quality using the LTR Assembly Index (LAI; Ou et al., 2018), which evaluates the completeness and continuity of long terminal repeat (LTR) retrotransposons (**Table 1**). LAI values > 20 are considered reference quality assemblies and indicate highly accurate and contiguous assembly of repetitive regions. The LAI for our assembly was 19.72.

### 3.2 Annotation

To facilitate *de novo* gene annotation, 10.3 M PacBio IsoSeq HiFi reads were produced from roots, stems, leaves, and whole seedlings that were desegmented into 80.5 M high-quality, full length, non-chimeric reads with an average read length of 1,607 bp. Annotation with Braker3 (Gabriel et al., 2024) identified 41,428 gene models, including alternative splicing variants. For comparison, Jaggi et al. (2025) reported 41,186 gene models for *C. sosnowskyi*, a related AAGG tetraploid species, while Rey et al. (2024) reported a range of 43,733 - 48,564 models for the more distantly related AABB tetraploid species *C. quinoa.* The average gene length was 4,215 bp, while the longest coding sequence was 16,491 bp. The total gene space (total gene length/total chromosome length) was 19.0%, while the total coding space occupied 6.4% of the total genome (**Supplementary Table 1**). As anticipated, gene density was highest at the chromosome ends and lowest in the putative centromeric regions (**Figure 1A**). BUSCO analysis of the predicted protein and transcript-coding gene models, assessed against the embryophyta and eudicot conserved ortholog datasets, identified 98.9% and 97.7% complete genes, respectively. These results were further supported by OMark analysis (Nevers et al., 2025), which reported 95.1% completeness and 94.4% lineage consistency. When the subgenomes were analyzed separately, 92.7% and 88.6% of COGs were identified as complete in the predicted protein models of the A subgenome using the embryophyta and eudicot datasets, respectively. Similarly, 91.0% and 88.6% completeness were identified in the corresponding transcript models. In contrast, the I subgenome showed lower completeness, with 80.5% and 81.3% of COGs identified as complete in the protein models using the embryophyta and eudicot datasets, respectively, and 80.2% and 81.1% completeness in the corresponding transcript models (**Supplementary Figure S4**). These results suggest that, although the annotation is sufficient for the comparative genomic analyses presented here, the I subgenome annotation could likely be improved with additional evidence-based refinement and curation.

### 3.3 Subgenome assignment

**Figure 2:**
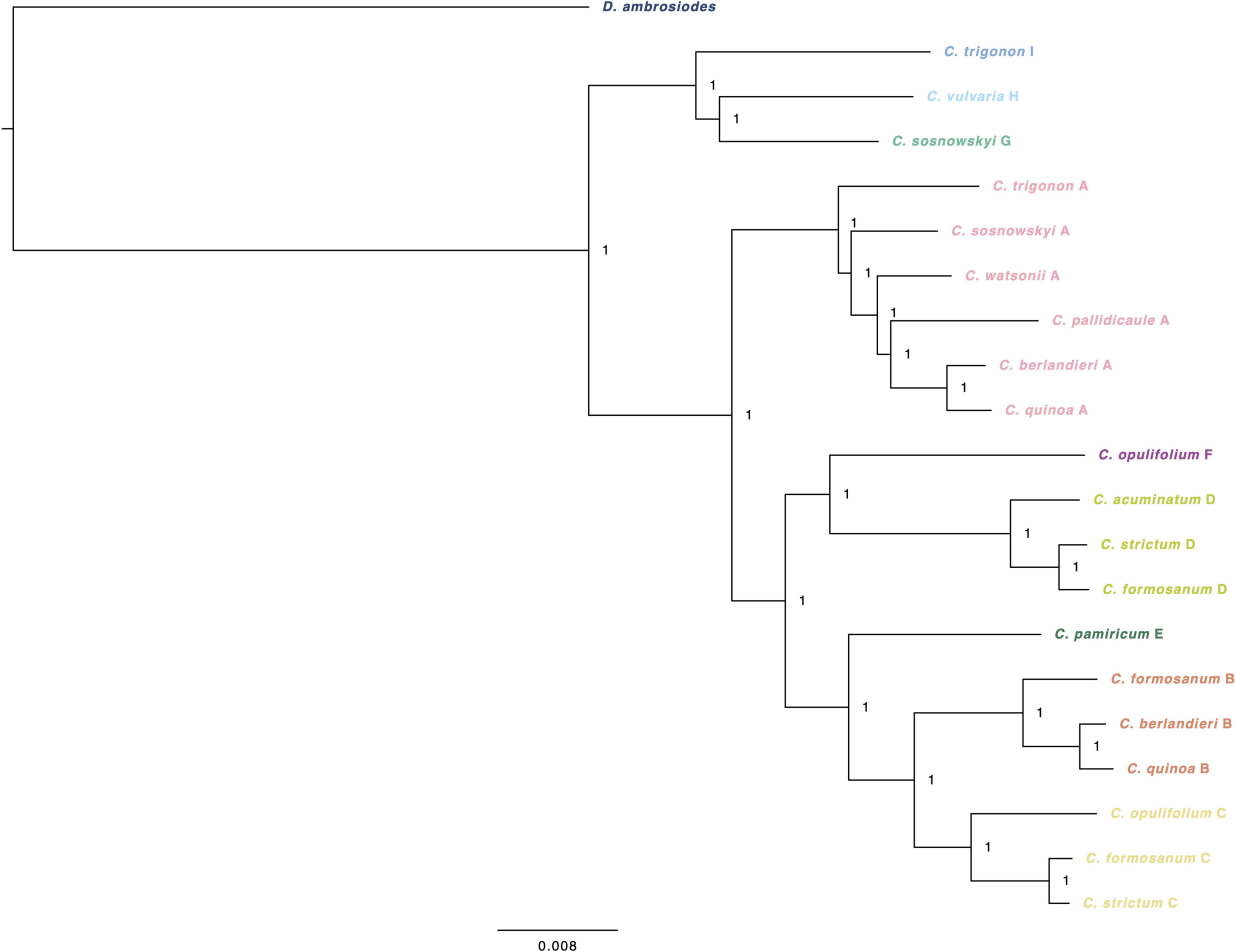
Subgenome phylogenetic tree based on OrthoFinder2 using the multiple sequence alignment argument with *Dysphania ambrosioides* (epazote) as the outgroup. Subgenome identity is indicated by the letter following each species name. MSA bootstrap values are shown at each node. The scale bar represents substitutions per site.

To assign each *C. trigonon* chromosome to a specific chromosome number (1–9) and known subgenome (A–H), we aligned the chromosomes against a reference panel consisting of chromosomes 1–9 from all eight subgenomes (Jaggi et al., 2025; **Figure 1B**). Chromosome identity and subgenome designation were determined based on overall alignment patterns, including sequence similarity, collinearity, and the proportion of aligned sequence to each reference chromosome. This analysis clearly identified nine chromosomes as pertaining to the A subgenome type, while the remaining nine chromosomes showed synteny with both the G and H subgenome types. Using OrthoFinder2 (Emms and Kelly, 2019) we investigated the phylogenetic relationship of the *C. trigonon* subgenomes relative to representatives of each subgenome (A–H) from other fully sequenced *Chenopodium* species outlined in **Supplementary Table 2**, including *C. quinoa* (AABB), *C. berlandieri* Moq. (AABB), *C. pallidicaule* Allen (AA), *C. watsonii* A. Nelson (AA), *C. sosnowskyi* (AAGG), *C. formosanum* Koidz. (BBCCDD), *C. opulifolium* (BBCCFF), *C. strictum* (CCDD), *C. acuminatum* Willd. (DD), *C. pamiricum* Iljin (EE) and *C. vulvaria* L. (HH). We identified 28,737 orthologous gene families across the 13 species. The reconstructed OrthoFinder2 phylogeny (**Figure 2**) indicates that *C. trigonon* is an allotetraploid, with its two subgenomes resolving into distinct and well-supported clades corresponding to the A and G/H genome lineages. The A subgenome of *C. trigonon* clusters within the broader A-genome clade but is distinct from other A-derived tetraploids, including the Eurasian AAGG tetraploid *C. sosnowskyi*. The second subgenome groups within the G/H lineage and is recovered as a sister group to the sampled G and H genome representatives. The placement of both subgenomes as sisters to their respective clades, and distinct from the corresponding subgenomes of *C. sosnowskyi*, supports an independent polyploidization event for *C. trigonon*. While branch lengths suggest substantial divergence, they do not allow strong inference regarding the relative timing of these events. Given this phylogenetic distinctness from known G and H subgenomes, together with its distinct repeat landscape (see Repeat Composition section), we tentatively designate this lineage as the “I” genome pending broader sampling of relatives. However, we note that this designation is provisional, and the observed pattern may also reflect divergence within a G/H-like lineage rather than a fully distinct genomic type and will require additional sampling to resolve its evolutionary origin.

### 3.4 Synteny

**Figure 3:**
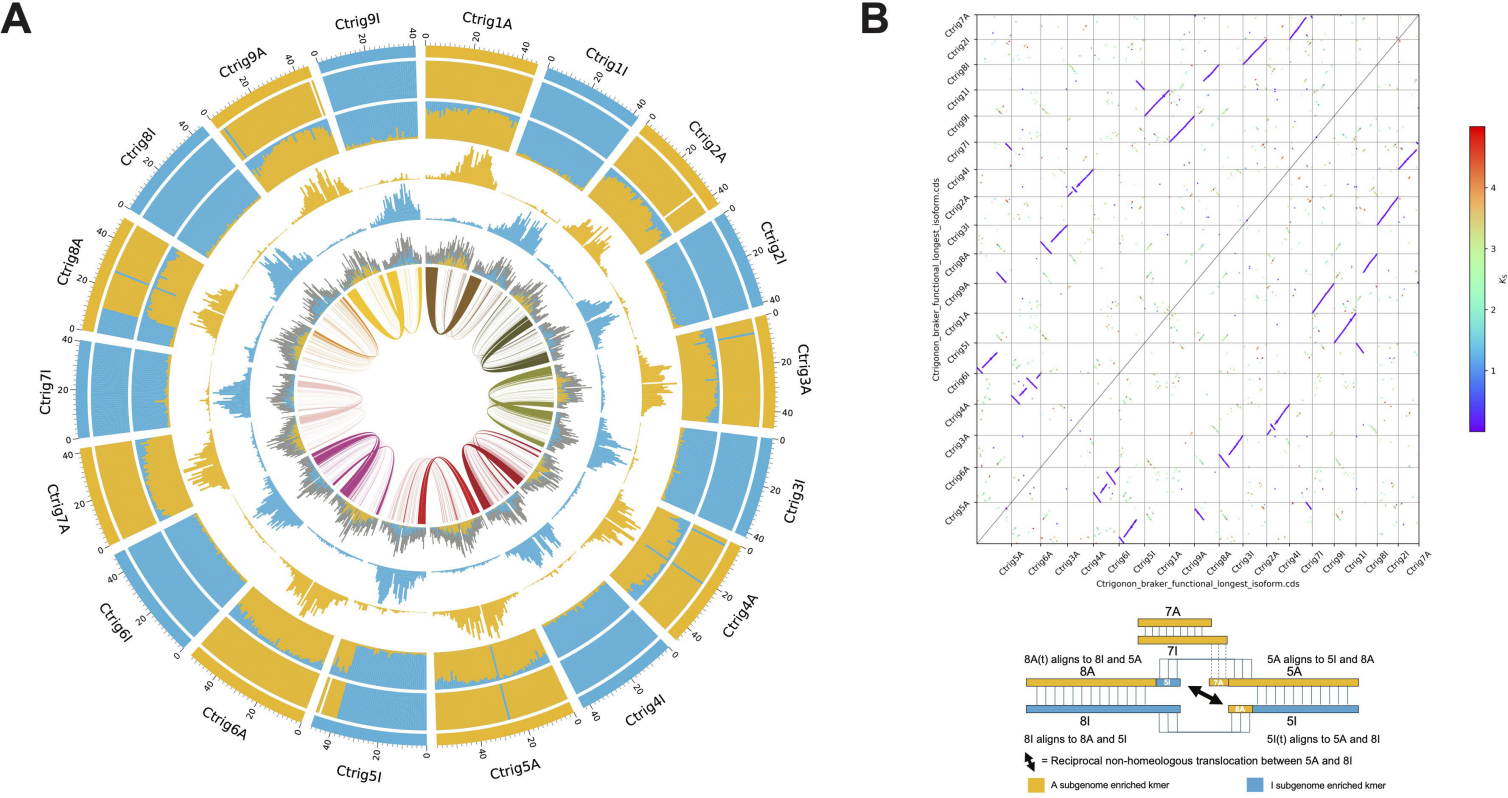
Subgenome assignment and structural relationships between A and I subgenomes. **(A)** Circos representation of SubPhaser subgenome assignments. In order starting from the outside, tracks represent 1) k-means–based subgenome assignment, 2) enrichment of subgenome-specific k-mers (colored regions indicate significant enrichment; white indicates non-significant windows), 3) relative proportion of subgenome-specific k-mers, 4) absolute counts of A- and I-specific k-mer sets, 5) LTR retrotransposon (LTR-RT) density (subgenome-colored regions indicate significant enrichment; gray indicates non-specific density), and 6) homoeologous blocks between subgenomes. All statistics were calculated in 1-Mb sliding windows. **(B)** Ks-based self–self synteny dot plot of *C. trigonon*. Collinear anchor gene pairs identified by wgd2 from a self-comparison of longest-isoform CDS sequences are colored by synonymous substitution rate (Ks). The inset illustrates an apparent reciprocal non-homeologous translocation between chromosomes 5A and 8I and an additional translocation event involving chromosomes 7A and 5A.

In accordance with the nomenclature conventions established by Jaggi et al. (2025), the chromosomes were oriented and renamed in agreement with their corresponding ortholog (chr1–9) and genome designations (A or I). The designation of the chromosomes to specific subgenomes was further validated using SubPhaser, which leverages differences in repetitive elements and k-mer composition to classify chromosomes into subgenomes (**Figure 3A**). All chromosomes displayed clear subgenome-specific k-mer enrichment consistent with their A and I subgenome assignments. Interestingly, Ctrig8A and Ctrig5I exhibited localized deviations in k-mer enrichment patterns, suggestive of potential non-homoeologous exchange or regions of reduced subgenome divergence in these chromosomes. Visualization of the relationships between the A and I subgenomes using self–self synteny dot plots confirmed the Ctrig 8A:5I translocation as well as an additional simple terminal translocation between Ctrig 5A and 7I (**Figure 3B**). It is unlikely that these rearrangements are assembly artifacts, as the Hi-C data show a strong intrachromosomal interaction signal supporting these rearrangements (**Supplementary Figure S1**).

**Figure 4:**
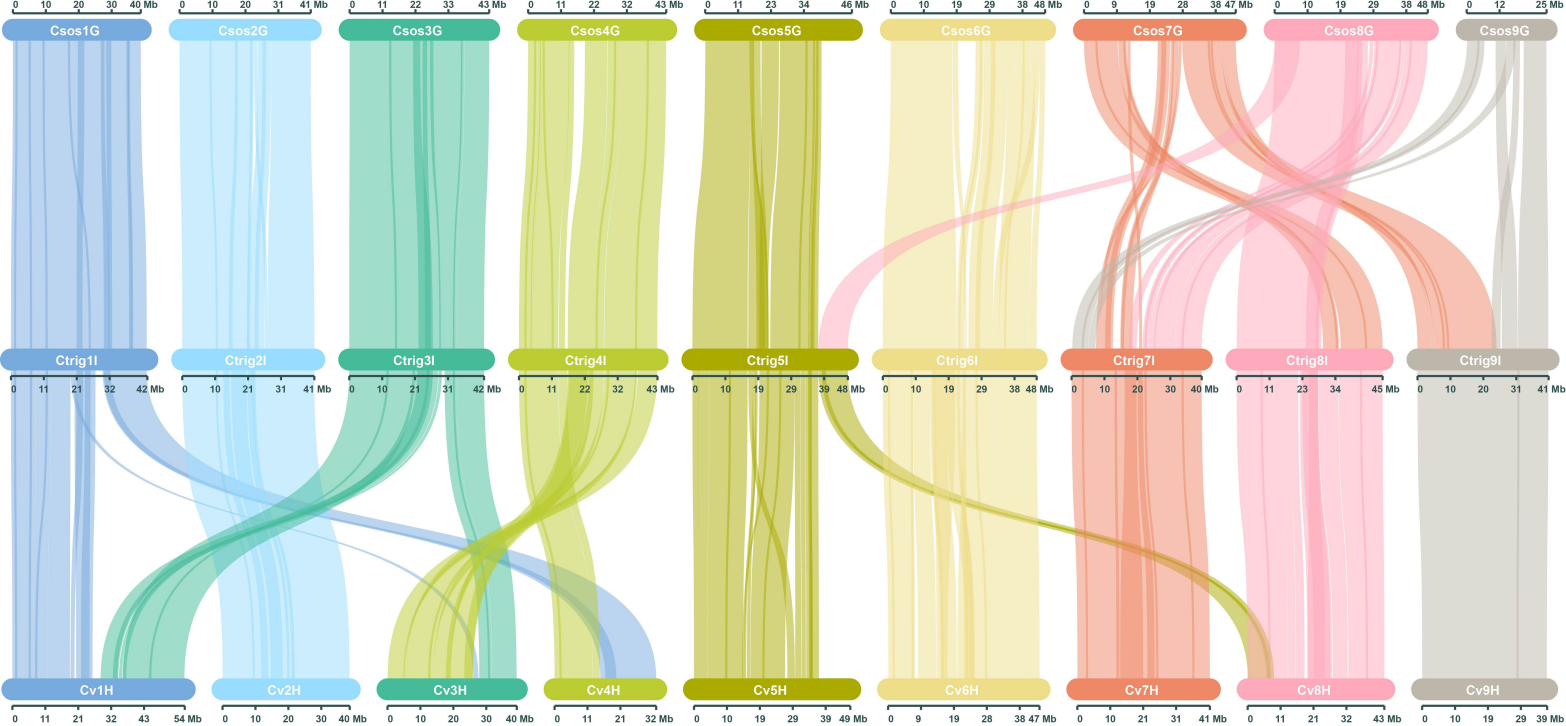
Syntenic gene blocks among the G subgenome of *C. sosnowskyi*, the I subgenome of *C. trigonon*, and the H subgenome of *C. vulvaria*.

Syntenic relationships among the G, H, and I subgenomes of *C. sosnowskyi*, *C. vulvaria*, and C*. trigonon* were examined using SynVisio (Bandi et al., 2022), based on syntenic blocks identified with MCScanX (**Figure 4**). Two major groups of complex structural rearrangements were identified. The first involves extensive interchromosomal exchanges among chromosomes 1, 3, and 4 of *C. trigonon* and *C. vulvaria*. The second comprises multiple rearrangements among chromosomes 7–9 of *C. trigonon* and *C. sosnowskyi*. In contrast, chromosomes 2 and 6 exhibit strong collinearity and high syntenic conservation across all three subgenomes. Notably, there is a reciprocal translocation shared between chromosomes 8G and 8H of *C. sosnowskyi* and *C. vulvaria*, respectively, relative to chromosome 5I of *C. trigonon*. The pronounced structural divergence of the *C. trigonon* I subgenome relative to the G and H subgenomes further supports its recognition as a distinct subgenome type.

### 3.5 Repeat composition

**Figure 5:**
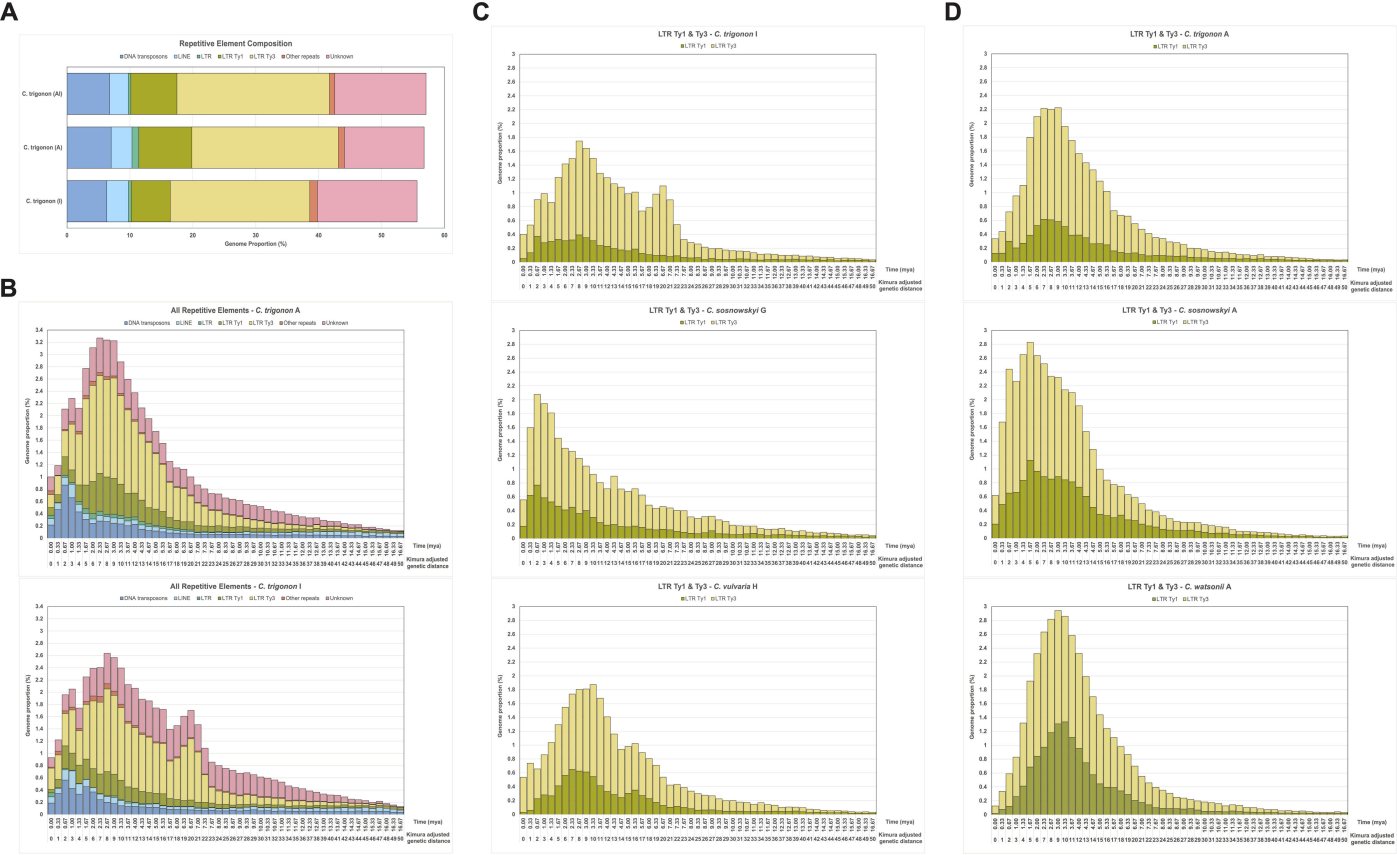
Repeat composition and LTR retrotransposon landscapes in *C. trigonon* and related subgenomes. **(A)** Abundance of major repetitive element categories in the whole genome and in the A and I subgenomes of *C. trigonon*. **(B)** LTR Ty1- and Ty3-retrotransposon landscape plots for the A and I subgenomes of *C. trigonon*. The y-axis shows TE abundance as a percentage of the genome. The x-axis represents sequence divergence (CpG-adjusted Kimura distance) from consensus sequences, with corresponding estimates of insertion time calculated using LTR substitution rate estimates from *Arabidopsis thaliana*. **(C)** LTR Ty1- and Ty3-retrotransposon landscapes for the I subgenome of *C. trigonon* compared with the G and H subgenomes of *C. sosnowskyi* and *C. vulvaria*, respectively. **(D)** LTR Ty1- and Ty3-retrotransposon landscapes for the A subgenomes of *C. trigonon*, *C. sosnowskyi*, and *C. watsonii*.

Approximately 58% of the *C. trigonon* genome was classified as repetitive by Earl Grey (**Figure 5A**, **Supplementary Table 3**), with long-terminal repeats (LTRs) being the most abundant repetitive element (32%) of the assembly. LTRs are the most abundant repeat element found in plants, and their abundance correlates with genome size (Tenaillon et al., 2010; Jaggi et al., 2025). In *C. trigonon* the A subgenome (433.8 Mb) is larger than the I subgenome (391.8 Mb), even though the number of non-repetitive bases is similar in both subgenomes (174 Mb and 170 Mb in A and I, respectively), supporting the hypothesis that the repetitive fraction may contribute to the increased size of the A subgenome. Indeed, there is an increase in LTR content in the A subgenome relative to the I subgenome (33% vs. 29%), with an increase in both Ty1- and Ty3-retrotransposons (**Figure 5A**). The genome fraction of Ty3-retrotransposons (A: 23.3% and I: 22.1%) and Ty1-retrotransposons (A: 8.4% and I: 6.2%) are consistent with, and fall within, the expected ranges of 12.6–35.5% and 4.8–15.2% previously estimated for *Chenopodium* (Jaggi et al., 2025). Repeat landscape plots of the *C. trigonon* A and I subgenomes show broadly overlapping peaks of LTR Ty1-retrotransposon abundance centered around k=7 (~1.5–4.0 mya; **Figure 5B**), indicating broadly similar recent transposable element dynamics between the subgenomes. The elevated Ty1-retrotransposon content in A may reflect more extensive historical proliferation and/or greater retention of these elements, and may contribute to the larger LTR fraction observed in the A subgenome. LTR Ty3-retrotransposon landscape plots differ more substantially between the subgenomes, specifically, the A subgenome exhibits a single major abundance peak centered near k=7, whereas the I subgenome shows a broader distribution of older elements, including signal overlapping the k=7 peak observed in the A subgenome and a more diffuse signal around k=20 (~6.0–7.3 mya) that appears more prominent in the I subgenome (**Figure 5B**). This older peak may indicate a Ty3-retrotransposon expansion that occurred prior to polyploidization. Alternatively, these differences may reflect differential retention or loss of older elements between subgenomes, rather than a distinct ancestral expansion event. If a comparable older expansion also occurred in the A genome, it may no longer be detectable (repeat landscapes reflect only insertions that have been retained). The potential influence of assembly and annotation biases also cannot be excluded.

Given the close relationship between the G, H and I subgenomes (**Figure 4**), we investigated the repeat dynamics between the I subgenome of *C. trigonon*, the G subgenome of *C. sosnowskyi*, an AAGG tetraploid, and the H subgenome of *C. vulvaria*, an HH diploid (**Figure 5C**). Both LTR Ty1- and Ty3-retrotransposons showed distinct signatures across G, H, and I subgenomes of the three species, including distinctive shifts in the number and relative timing of peaks (**Figure 5C**). The *C. trigonon* I subgenome contains a higher proportion of Ty3-retrotransposons and DNA transposons (with a slight decrease in Ty1-retrotransposons), resulting in a larger fraction of the genome being classified as repetitive (55.6%) compared to *C. sosnowskyi* G (49.3%) and *C. vulvaria* H (53%).

Similarly, comparison of A subgenomes of *C. trigonon*, *C. sosnowskyi* and North American AA diploid *C. watsonii* show that transposable element dynamics of Ty1- and Ty3-retrotransposons differ in both magnitude and temporal distribution (**Figure 5D**). In this comparison, *C. sosnowskyi* and *C. watsonii* exhibit notably prominent peaks of Ty1-retrotransposons. Consistent with this pattern, Ty1-retrotransposons comprise a substantially larger proportion of the A subgenomes of *C. sosnowskyi* (15%) and *C. watsonii* (14.4%) than of *C. trigonon* (8.4%). This elevated Ty1-retrotransposon abundance largely accounts for the higher repetitive fraction observed in the A subgenomes of *C. sosnowskyi* (61.4%) and *C. watsonii* (62.7%) compared with *C. trigonon* (56.8%) and likely contributes substantially to the larger A subgenome sizes of *C. sosnowskyi* (520.0 Mb) and *C. watsonii* (514.4 Mb) relative to *C. trigonon* (433.8 Mb). Taken together, the analysis of repetitive elements suggests that LTR retroelements have played major roles in subgenome differentiation between *C. trigonon* and *Chenopodium* species with similar subgenomes.

### 3.6 Allopolyploid origins inferred from Ks distributions

**Figure 6:**
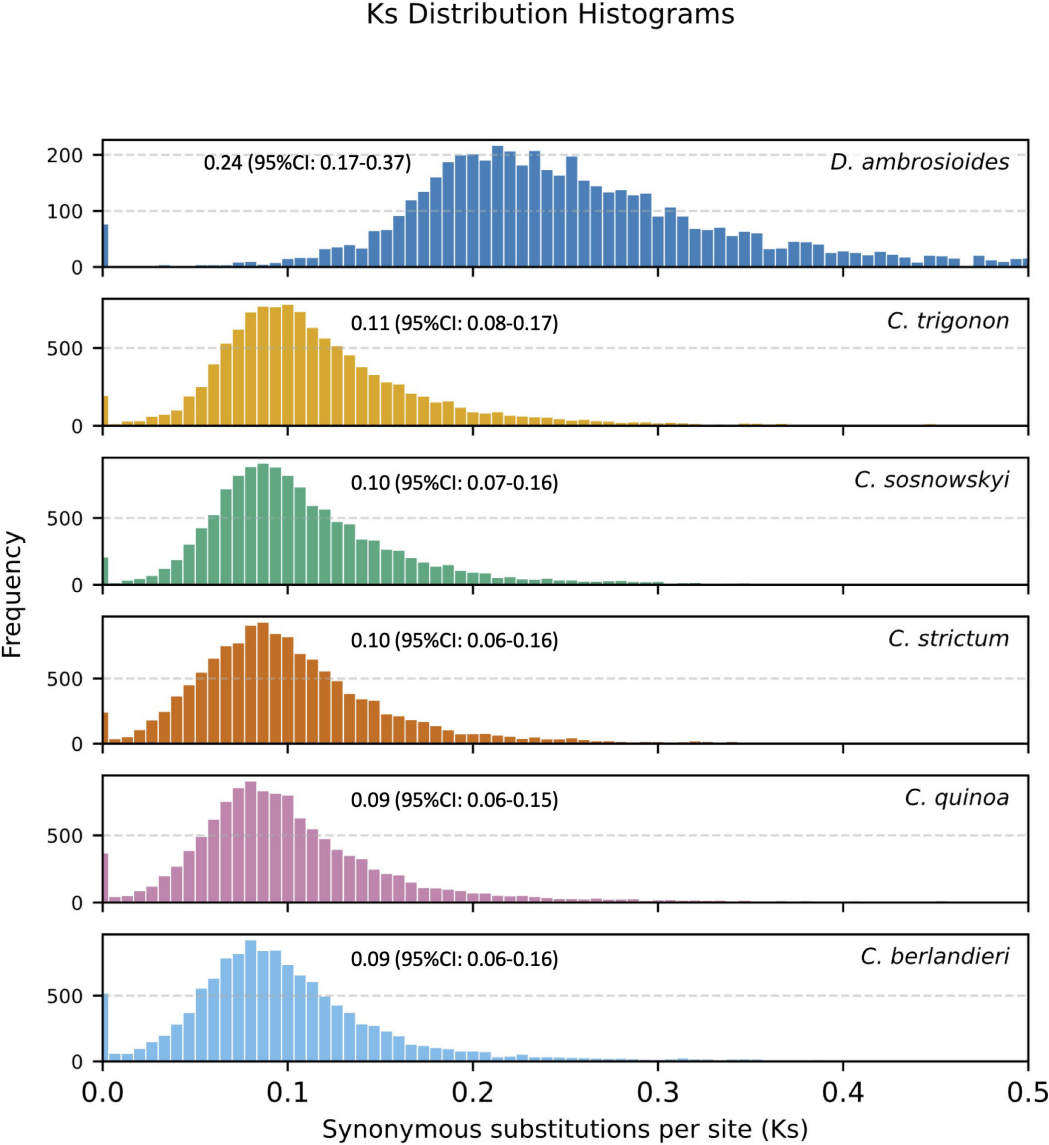
Distribution of synonymous substitution rates (Ks) among homologous gene pairs in *C. trigonon* and related tetraploid *Chenopodium* species. Histograms show the frequency of Ks values for collinear homologous gene pairs identified within each species. Median Ks values and 95% confidence intervals are indicated above each distribution. The x-axis represents synonymous substitutions per site (Ks), and the y-axis shows gene pair frequency.

To evaluate whether *C. trigonon* experienced a distinct allopolyploidization event, we compared synonymous substitution (Ks) distributions of anchor gene pairs using WGD2 (Chen et al., 2024) across *C. trigonon* and other tetraploid *Chenopodium* species, including *C. sosnowskyi* (AAGG), *C. strictum* (CCDD), *C. quinoa* (AABB), and *C. berlandieri* (AABB). The paleotetraploid amaranthaceous species *Dysphania ambrosioides* (L.) Mosyakin & Clemants was included for comparison (**Figure 6**).

The Ks distribution for *D. ambrosioides* exhibits a clearly older peak (Ks ≈ 0.24; 95% CI: 0.17–0.37), consistent with a more ancient polyploidization (likely allopolyploidization) event relative to *Chenopodium*. In contrast, all *Chenopodium* species displayed a prominent and largely overlapping Ks peak centered between 0.09 and 0.11. Specifically, peak Ks values occur at 0.11 for *C. trigonon* (95% CI: 0.08–0.17), 0.10 for *C. sosnowskyi* (0.07–0.16) and *C. strictum* (0.06–0.16), and 0.09 for the New World species *C. quinoa* (0.06–0.15) and *C. berlandieri* (0.06–0.16). Although the subgenome compositions of these tetraploids suggest independent allopolyploid origins, the near-coincident Ks peaks imply that these events occurred within a relatively narrow evolutionary window – suggestive of a scenario in which multiple polyploidization events arose during a period of intense hybridization and diversification within the genus (Mandák et al., 2026). While the slightly later Ks peak in *C. trigonon* could suggest marginally greater age relative to the other taxa, the overlapping confidence intervals preclude strong chronological inference.

To assess whether ancient whole-genome duplication (WGD) events are detectable within the A and I subgenomes, we generated Ks distributions for paralogous gene pairs using WGD v2. Homologous gene pairs were identified with DIAMOND (wgd dmd), and synonymous substitution rates were estimated with wgd ksd. The Ks distribution of the full allotetraploid genome (AAII) shows a pronounced peak at low Ks values, consistent with the recent allopolyploidization event (**Figure 6**). In contrast, the Ks distributions for the A (A vs. A) and I (I vs. I) subgenomes lack distinct secondary peaks at higher Ks values (**Supplementary Figure S5**). Instead, both subgenomes exhibit a strong enrichment of low-Ks pairs and a broad, gradually declining tail, a pattern consistent with ongoing small-scale duplication and gene turnover rather than a retained signal of an older WGD. We note that the absence of a clear Ks peak does not exclude the possibility of older duplication events. Indeed, the ancient WGD signal may be obscured by extensive diploidization, fractionation, and sequence divergence, as well as saturation of synonymous substitutions at higher Ks values, which can erode or eliminate recognizable peaks over time.

### 3.7 Chenopodium baccatum

To investigate whether the A and I genomes are present in other Australian *Chenopodium* species, we generated a PacBio HiFi draft assembly of *C. baccatum* Labill., a putative polyploid woody perennial shrub endemic to arid and semi-arid regions of Australia. The draft assembly consisted of 974 contigs totaling 1.958 Mb with an N50 of 40 Mb (**Supplementary Table 4**). Primary contigs were mapped to a reference chromosome panel comprising chromosomes 1–9 from all known genome types (A–H), as well as the A and putative I genomes of *C. trigonon* (**Supplementary Figure S6A**). The contigs mapped almost exclusively to the A (49.4%) and I (47.4%) subgenomes of *C. trigonon*, with minimal alignment to the A or G subgenomes of *C. sosnowskyi* or the H subgenome of *C. vulvaria* (**Supplementary Figure S6B**). Interestingly, the alignment pattern was consistent with an alloautooctoploid genomic constitution (2n = 8x = 72; putatively AAAAIIII), likely derived from genome doubling of an ancestral AAII tetraploid lineage. Flow cytometry estimates further support the octoploid status of *C. baccatum*, with a genome size of 4.4–4.5 pg, approximately double that of *C. trigonon* (2.03 pg; P. Trávníček, personal communication, Czech University of Life Sciences). Because *C. baccatum* is also endemic to Australia, this shared A and I genomic composition raises the possibility that an ancestral AI polyploidization event, or repeated hybridization among related A- and I-like progenitors, contributed to the diversification of *Chenopodium* within the Australian continent. While this scenario remains to be tested through expanded phylogenomic sampling and explicit temporal analyses, the combined phylogenetic, syntenic, and repeat landscape evidence supports the view that Australian taxa represent a distinct and evolutionarily independent genomic lineage within the genus.

## 4 Conclusions

In summary, differences in genome size between *C. trigonon* (825.6 Mb) and *C. sosnowskyi* (900.6 Mb) are primarily attributable to variation in repetitive content, particularly that of LTR retrotransposons. The larger genome of *C. sosnowskyi* is associated with greater overall LTR accumulation, including elevated Ty1-retrotransposon content in the A subgenome and subgenome-specific differences in Ty3-retrotransposon and DNA transposon abundance. Phylogenetic and syntenic analyses support the interpretation of *C. trigonon* as a distinct AAII tetraploid lineage derived from an independent hybridization event involving unsampled or extinct diploid progenitors. Although Ks distributions among *Chenopodium* species are broadly overlapping and do not indicate a clearly older polyploidization event in *C. trigonon*, the phylogenetic positions of its A and I subgenomes within their respective clades are consistent with early divergence from other tetraploid lineages. The geographic separation between *C. trigonon* (Australia) and *C. sosnowskyi* (Eurasia) further supports independent origins. Differences in transposable element composition and landscape profiles between species, interpreted cautiously given the limitations of divergence-based dating, are concordant with independent evolutionary trajectories following polyploid formation.

## Data Availability

The datasets presented in this study can be found in online repositories. The names of the repository/repositories and accession number(s) can be found in the article/**Supplementary Material**.
