# Supplementary material for: Chromosome-level genome assembly of the Australian Chenopodium trigonon reveals a new subgenome type
Source: Front Plant Sci. 2026 Jun 11;17:1841078. doi: 10.3389/fpls.2026.1841078 (PMC13294208; doi:10.3389/fpls.2026.1841078)

Supplementary Material

Supplemental Tables

Supplemental Table S1. Braker3 annotation statistics for all predicted gene models and for longest isoforms only.

	Complete Annotation	Longest Isoform Only
**Number of genes**	37184	37184
**Number of mRNAs**	41428	37184
**Number of single exon genes**	8586	8694
**Number of exons**	226998	196734
**Number of CDS**	41428	37184
**Number of introns**	185570	159550
**Longest gene (bp)**	136061	136061
**Longest CDS (bp)**	16491	16491
**Shortest intron (bp)**	21	27
**Mean gene length (bp)**	4215	4190
**Mean exon length (bp)**	234	237
**Mean mRNA length (bp)**	4330	4190
**Mean exons per CDS**	5.5	5.3
**Mean intron length (bp)**	680	684
**Mean introns per mRNA**	4.5	4.3
**Total CDS length (bp)**	53158334	46659305
**Total gene length (bp)**	156723531	155784640
**Total mRNA length (bp)**	179376311	155784640
**Total intron length (bp)**	126217977	109125335

Supplemental Table S2. *Chenopodium* species genome and collection information.

Species	Accession	Ploidy (genome)	Genome Size (bp)	Collection Location
C. acuminatum	BYU 2013	2x (DD)	439,645,778	Altaiskii Krai, Mamontovinskii, Russia
C. baccatum	BYU 2414	8x (AAAAIIII)	1,958,361,148	Rockingham, Western Australia, Australia
C. berlandieri ssp. *nuttaliae*	BYU 1484	4x (AABB)	1,295,514,343	Atlixco Municipality, Puebla, Mexico
C. formosanum	BYU 2081-2083	6x (BBCCDD)	1,630,219,857	Wutai Twp. Pingtung Co., Taiwan
C. opulifolium	BYU 2219	6x (BBCCFF)	1,851,076,175	Salamanca, Castilla y Leon, Spain
C. pallidicaule	BYU 1652	2x (AA)	362,509,632	La Paz, Bolivia
C. pamiricum	BYU 2269, 1489/3	2x (EE)	542,061,588	Tyva Republic, Russia
C. quinoa	BYU 1637	4x (AABB)	1,326,337,151	Maule, Chile
C. sosnowskyi	BYU 2212	4x (AAGG)	926,508,239	West Azerbaijan, Iran
C. strictum	BYU 2104	4x (CCDD)	858,237,251	Prague Troja, Czech Republic
C. trigonon	BYU 2439	4x (AAII)	830,898,497	Swan Reach, Victoria, Australia
C. vulvaria	BYU 2205	4x (HH)	421,862,273	Budapest, Hungary
C. watsonii	BYU 873	2x (AA)	547,762,155	Yavapai, Arizona, USA

Supplemental Table S3. High-level repetitive element counts from EarlGrey for chromosome-scale assemblies of *C. trigonon* (AI, A, and I), *C. sosnowskyi* (AG, A, and G), *C. vulvaria*, and *C. watsonii*. Unplaced contigs were excluded from the analysis.

Species (Genome)	TE Classification	Coverage (bp)	Copy Number	% Genome Coverage	Genome Size (bp)	TE Family Count
***C. trigonon* (AI)**	DNA	55826411	67738	6.76	825555050	67738
	Rolling Circle	4721045	5797	0.57	825555050	5797
	Penelope	4485667	6233	0.54	825555050	6233
	LINE	24465024	32518	2.96	825555050	32518
	SINE	94313	191	0.01	825555050	191
	LTR	267601627	133531	32.41	825555050	133531
	Other	4329991	11530	0.52	825555050	11530
	Unclassified	119889726	319465	14.52	825555050	319464
	Non-Repeat	344141246	NA[a]	41.68	825555050	NA
***C. trigonon* (A)**	DNA	30411460	33726	7.01	433785699	33726
	Rolling Circle	2592229	3461	0.59	433785699	3461
	Penelope	2266308	2310	0.52	433785699	2310
	LINE	14333354	17193	3.30	433785699	17193
	SINE	223497	1464	0.05	433785699	1464
	LTR	142434434	69742	32.83	433785699	69742
	Other	11712710	13294	2.70	433785699	13294
	Unclassified	55007164	148392	12.68	433785699	148392
	Non-Repeat	174804543	NA	40.29	433785699	NA
***C. trigonon* (I)**	DNA	24754735	27790	6.31	391769351	27790
	Rolling Circle	1987996	2674	0.50	391769351	2674
	Penelope	3330264	4059	0.85	391769351	4059
	LINE	13499130	17179	3.44	391769351	17179
	SINE	191911	925	0.04	391769351	925
	LTR	114374559	56221	29.19	391769351	56221
	Other	2024585	4645	0.51	391769351	4645
	Unclassified	62158818	165192	15.86	391769351	165192
	Non-Repeat	169447353	NA	43.25	391769351	NA
***C. sosnowskyi* (AG)**	DNA	39712791	51685	4.40	900596145	51685
	Rolling Circle	7481371	9027	0.83	900596145	9027
	Penelope	2145214	4566	0.23	900596145	4566
	LINE	24117764	32350	2.67	900596145	32350
	SINE	269687	744	0.02	900596145	744
	LTR	364894907	162635	40.51	900596145	162635
	Other	6246868	16318	0.69	900596145	16318
	Unclassified	111366755	320072	12.36	900596145	320072
	Non-Repeat	344360788	NA	38.23	900596145	NA
C. sosnowskyi	DNA	23666133	29942	4.55	519961807	29942

**(A)**	Rolling Circle	3440958	4427	0.66	519961807	4427
	Penelope	669041	1748	0.12	519961807	1748
	LINE	12842400	13845	2.46	519961807	13845
	SINE	148213	297	0.02	519961807	297
	LTR	214990688	98485	41.34	519961807	98485
	Other	18838171	10152	3.62	519961807	10152
	Unclassified	65256263	166640	12.55	519961807	166640
	Non-Repeat	180109940	NA	34.63	519961807	NA
***C. sosnowskyi* (G)**	DNA	14100436	20676	3.70	380634338	20676
	Rolling Circle	1237374	1426	0.32	380634338	1426
	Penelope	1579507	2710	0.41	380634338	2710
	LINE	9432804	13998	2.47	380634338	13998
	SINE	50423	89	0.013	380634338	89
	LTR	105876295	60683	27.81	380634338	60683
	Other	27729263	9633	7.28	380634338	9633
	Unclassified	56544490	156443	14.85	380634338	156442
	Non-Repeat	164083746	NA	43.10	380634338	NA
***C. vulvaria* (H)**	DNA	19595221	27625	5.10	383631019	27625
	Rolling Circle	1647732	2096	0.42	383631019	2096
	Penelope	3042116	2888	0.79	383631019	2888
	LINE	9922446	12951	2.58	383631019	12951
	SINE	32943	54	0.01	383631019	54
	LTR	112978671	63583	29.44	383631019	63583
	Other	11454853	5269	2.98	383631019	5269
	Unclassified	56831021	156186	14.81	383631019	156186
	Non-Repeat	168126016	NA	43.82	383631019	NA
***C. watsonii* (A)**	DNA	29788455	34595	5.7905671	514430702	34595
	Rolling Circle	3532396	5801	0.6866612	514430702	5801
	Penelope	2786779	4136	0.5417210	514430702	4136
	LINE	16522680	17959	3.2118379	514430702	17959
	SINE	73601	207	0.0143073	514430702	207
	LTR	201418622	94579	39.1536938	514430702	94579
	Other	4276186	11187	0.8312463	514430702	11187
	Unclassified	69355232	169194	13.4819387	514430702	169194
	Non-Repeat	186676751	NA	36.2880268	514430702	NA

[a]NA, Not Applicable

Supplemental Table S4. Assembly statistics of the *Chenopodium baccatum* contig assembly.

Total length (bp)	Number of contigs	Largest contig	N50 (bp)	L50	N90 (bp)	L90
1,958,361,148	974	58,147,938	40,762,048	21	7,072,310	61

Supplemental Table S5. Sequencing resources and data availability. Sequencing data is deposited in the NCBI Sequence Read Archive (SRA) under BioProject ID PRJNA1428860. Species name, BioSample ID, experiment type, and corresponding NCBI SRA accession or genome assembly ID are provided. Genome assemblies for *C. trigonon* and *C. baccatum* are also available through the Comparative Genomics platform in CoGe (ID 69682 and 69854, respectively; CyVerse) and in the data download section of quinoaDB (http://quinoadb.org/).

Species	BioSample ID	Experiment	Genome ID or NCBI SRA#
C. trigonon	SAMN55974077	Genome Assembly	JBVTLR000000000
C. trigonon	SAMN55974077	WGS - PacBio HiFi	SRR37391188 - SRR37391189
C. trigonon	SAMN55974077	Hi-C - Illumina	SRR37391187
C. trigonon	SAMN55974077	IsoSeq - PacBio	SRR37391190
C. baccatum	SAMN55974706	WGS - PacBio HiFi	SRR37391185

Supplemental Figures

Supplemental Figure S1. Hi-C contact heatmap of the *C. trigonon* genome assembly. Genome-wide Hi-C interaction frequencies are shown at the contig level (green) and scaffold level (blue) for all 18 labeled pseudomolecules. Interaction intensity increases from white to red, with the scale bar measuring raw observed counts. Axes represent genomic coordinates (1 Mb resolution) across the assembled genome.

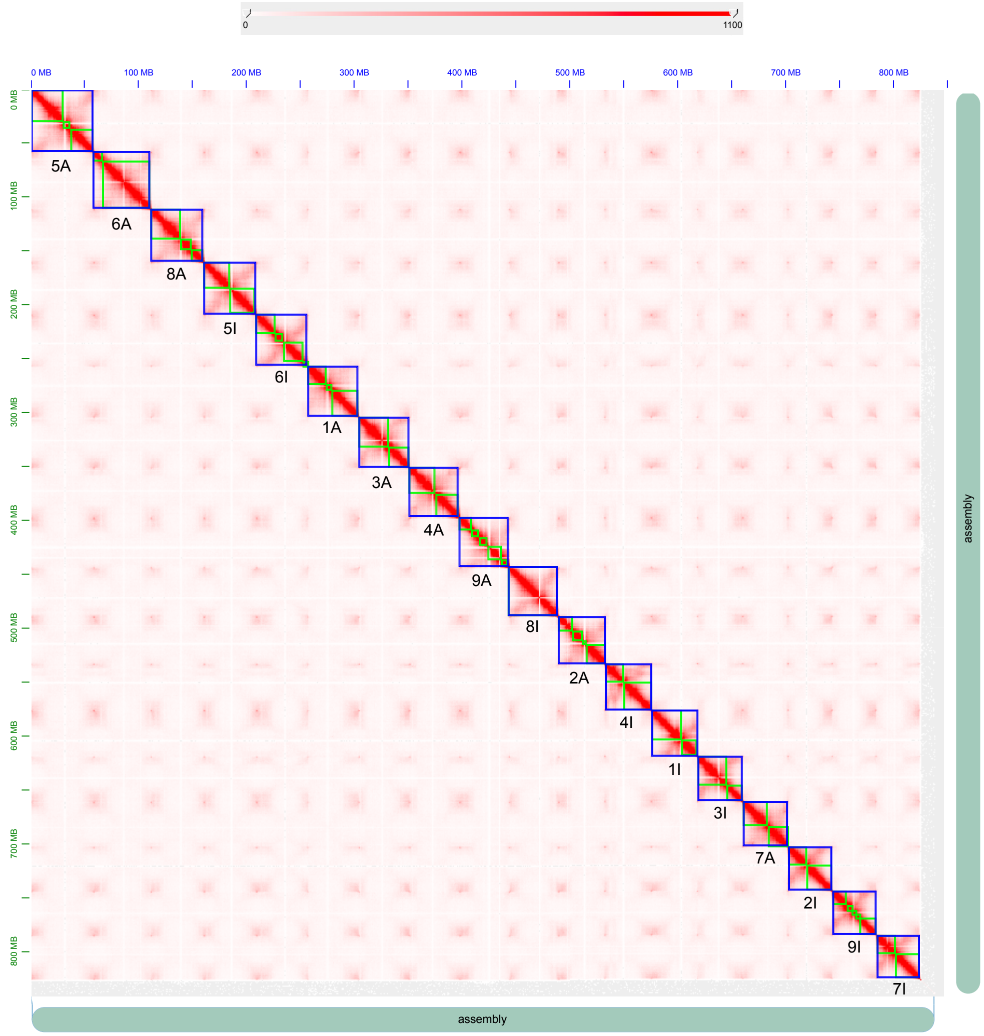

Supplemental Figure S2. Assembled and annotated chloroplast genome of *C. trigonon*. Circular map of the 152,126 bp chloroplast genome showing gene annotation and genome structure. Genes are colored by functional category. The large single-copy (LSC), small single-copy (SSC), and inverted repeat (IRa and IRb) regions are indicated. The inner gray histogram represents GC content across the genome.

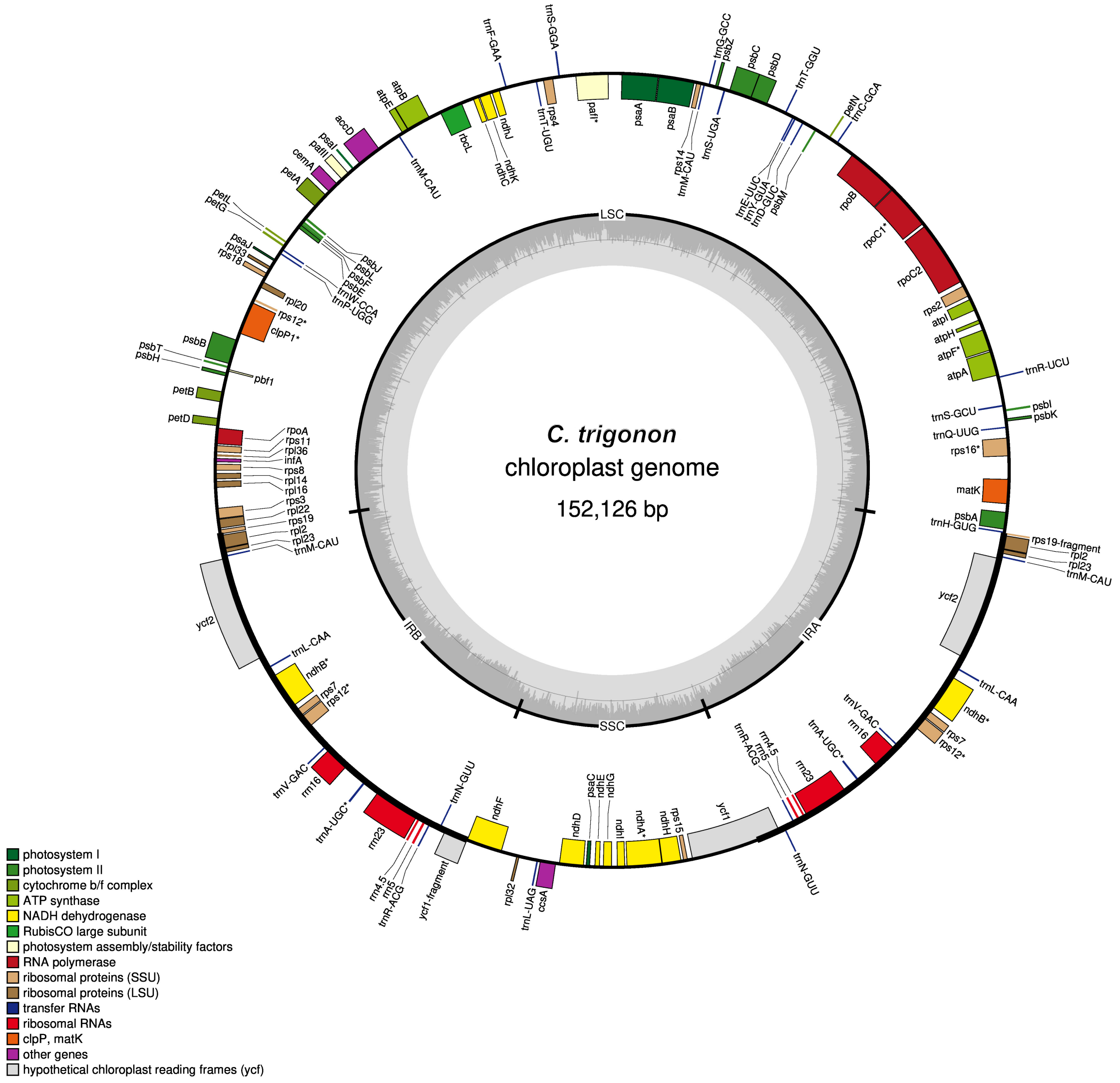

Supplemental Figure S3. Estimation of genome size and unique sequence content of the *C. trigonon* genome based on k-mer analysis estimated with GenomeScope 2.0.

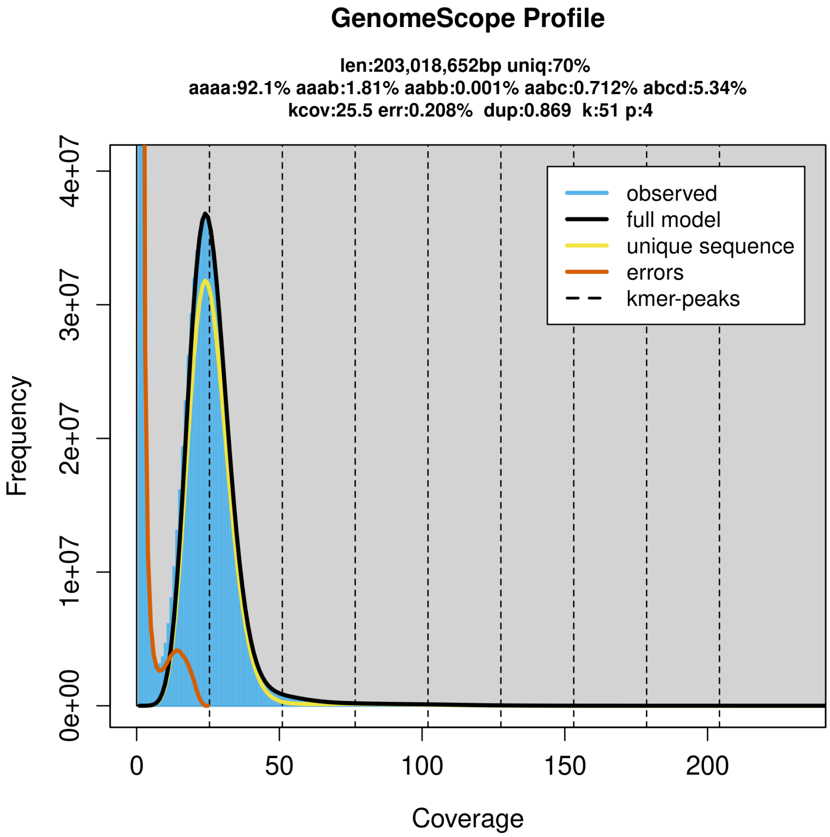

Supplemental Figure S4. BUSCO assessment of *C. trigonon* genome assembly and gene annotation completeness. **(A)** Completeness of the genome assembly, predicted protein sequences, and predicted transcript sequences for the combined assembly (AI) and the individual A and I subgenome assemblies, evaluated using the Embryophyta odb10 BUSCO dataset (n = 1,614). **(B)** Completeness of the genome assembly, predicted protein sequences, and predicted transcript sequences for the combined assembly (AI) and the individual A and I subgenome assemblies, evaluated using the Eudicots odb10 BUSCO dataset (n = 2,326). Bars indicate the percentage of complete (single-copy and duplicated), fragmented, and missing BUSCOs.

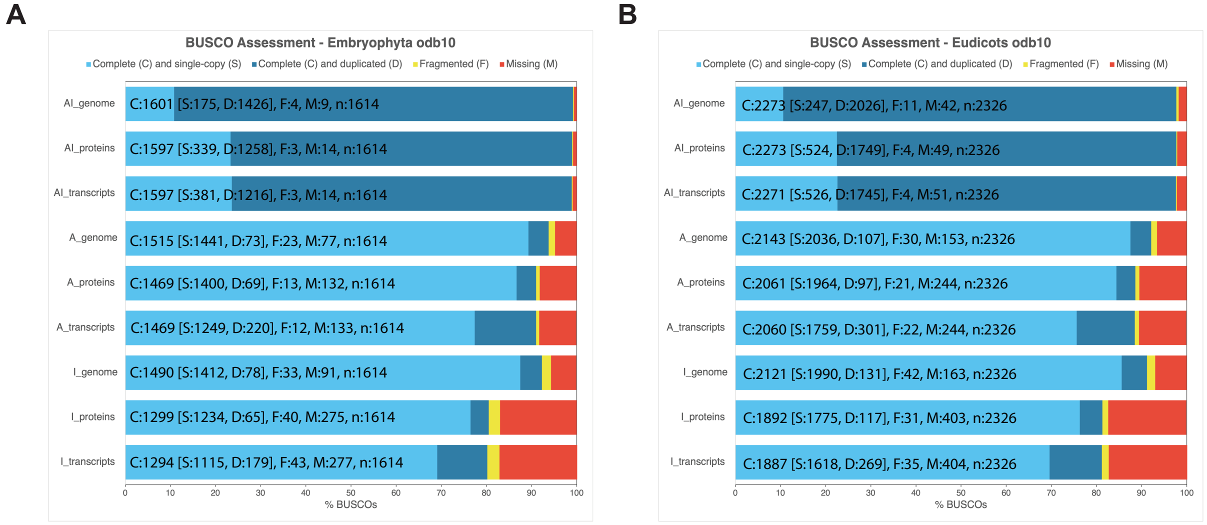

Supplemental Figure S5. Ks distributions for paralogous gene pairs in the A and I subgenomes of *C. trigonon* and the allotetraploid genome. **(A)** A subgenome (A vs. A), **(B)** I subgenome (I vs. I), and **(C)** the combined allotetraploid genome (AAII). Histograms show the frequency of paralogous gene pairs across Ks values, with black lines indicating smooth density curve. The allotetraploid genome (C) exhibits a pronounced peak at low Ks values, consistent with the recent allopolyploidization event (See Fig. 6). In contrast, the within-subgenome self comparisons (A, B) lack distinct secondary peaks at higher Ks values, indicating no detectable signal of an additional ancient whole-genome duplication. Instead, both subgenomes show a strong enrichment of low-Ks pairs and a broad, gradually declining distribution consistent with background small-scale duplication and gene turnover.

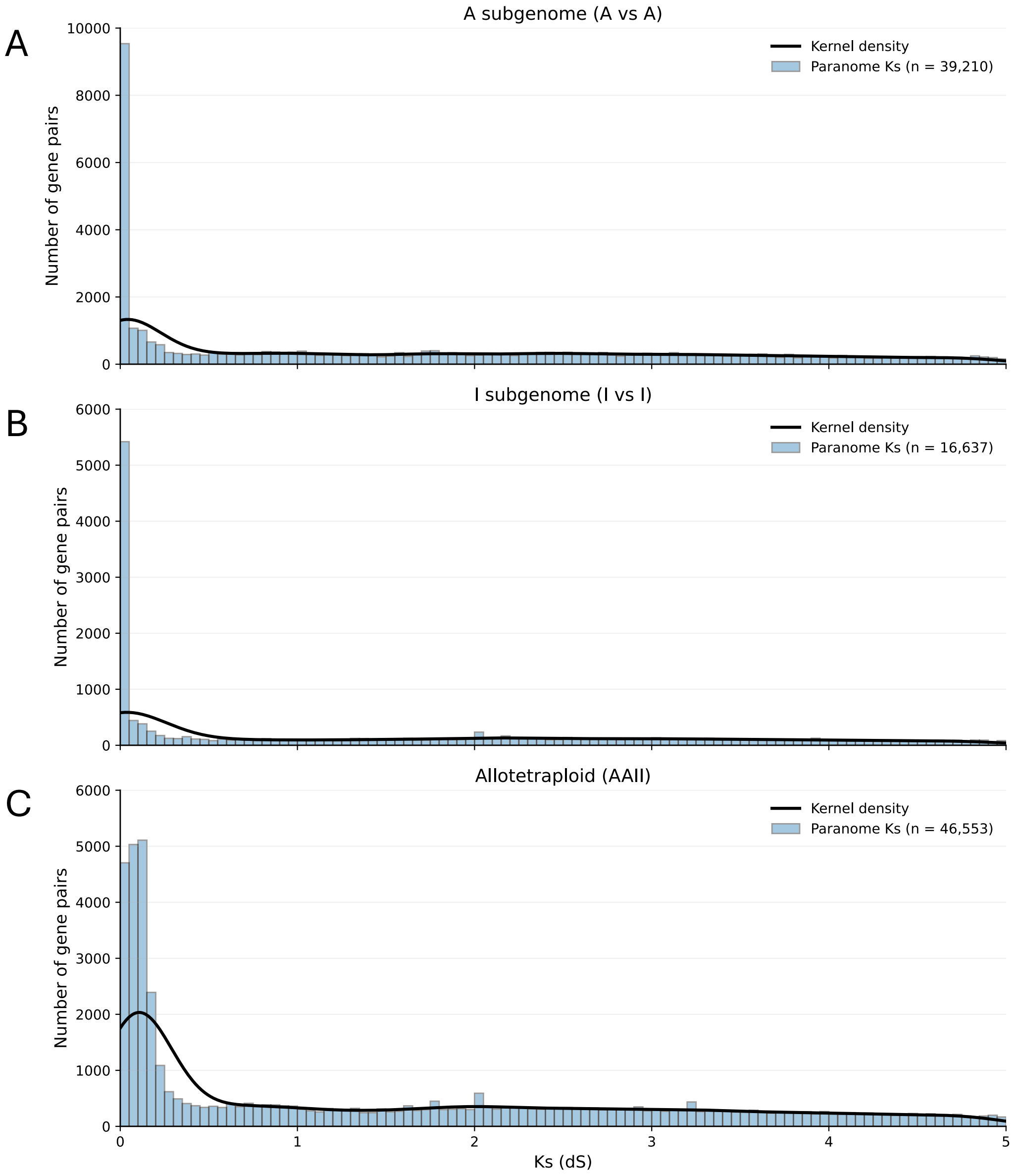

Supplemental Figure S6. Subgenome assignment of *Chenopodium baccatum* contigs. **(A)** Syntenic dot plot showing chromosome-scale correspondence between *C. baccatum* contigs and a panel of chromosomes from the A–I *Chenopodium* subgenomes. Subgenomes are ordered as A (*C. sosnowskyi*), A (*C. trigonon*), B, C, D, E, F, G, H, and I. **(B)** Pie chart showing the proportion of total *C. baccatum* contig length assigned to each *Chenopodium* subgenome based on the chromosome with the greatest aligned base-pair coverage for each contig. Subgenomes contributing <0.3% of the total assigned contig length (C, D, E, and G) were excluded for clarity.

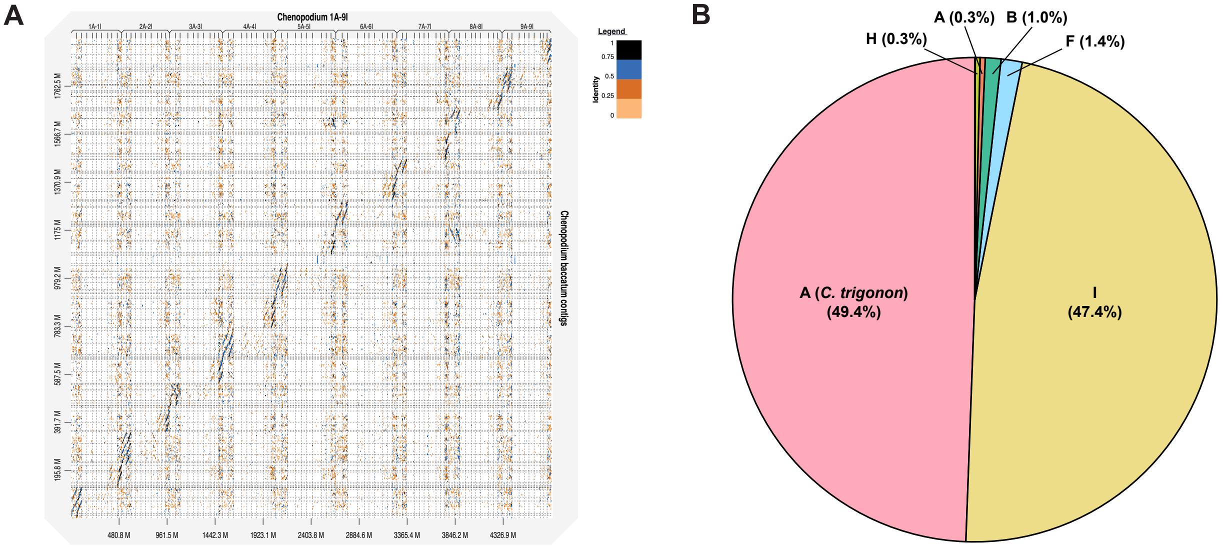

Supplemental Figure S7. Seed morphology of Australian *Chenopodium* species. **(A)** *C. trigonon* seeds ranging from agranular (left) to pericarpic (right). **(B)** *C. baccatum* seeds ranging from apericarpic (left) to berried (right). Scale bars: 1 mm (A) and 2 mm (B).

A B

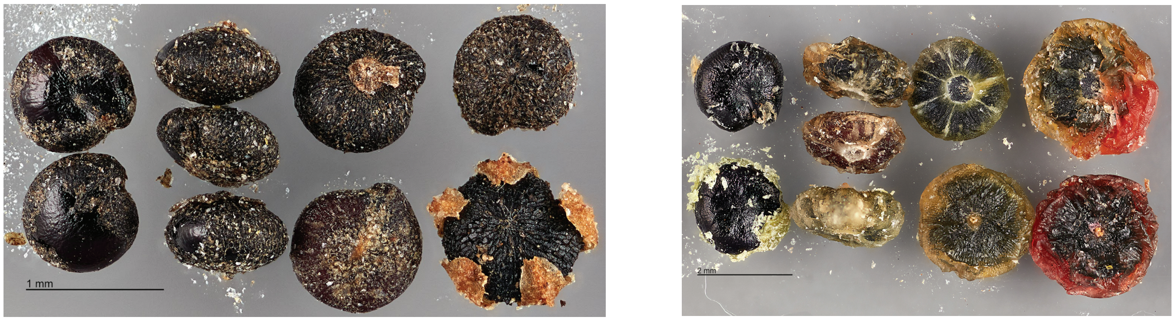

Supplement: Supplementary file 1 [file DataSheet1.pdf]
